# Aphasia

**Published:** 1883-07

**Authors:** H. R. Hopkins


BUFFALO

Medical and Surgical Journal.

Vol. XXII. JULY, 1883. No. 12.

### Original Communications.

### Aphasia.*

By H. R. Hopkins, M. D.

*Mr. President and Fellows:*

As an introduction to my remarks upon aphasia, I would invite your attention to the following case:

Mrs. C. C. F., of about 67 years of age, had been reared in an atmosphere of ease, wealth, and the most remarkable devotion; had been twice married, and worshipped by both her husbands, the latter having been the President of our nation. I had known of her health for years before she came under my personal observation, and knew that she had the usual trivial but absorbing ills of the idle and the wealthy. I was also not unfamiliar with the gossip of her neighbors, and had frequently heard it remarked that she was a woman of the most decided intemperance in the use of stimulants and narcotics, and of the most ungovernable temper.

During my service as her medical adviser, I was perfectly satisfied that there was not a word of truth in the rumor concerning her intemperance, but all sufficient grounds for the

* Read before the Buffalo Medical Club.

belief regarding the instability of her moods. I refer to this partly for the chance of bearing testimony to her good character, which was sadly slandered by rumor, and partly to emphasize the importance of certain mental states as signs of cerebral conditions antecendent to cerebral hemorrhage. Authorities upon lesions of the brain frequently mention uncertainty of gait, irritability of temper, and instability of purpose, as among the premonitory signs of cerebral hemorrhage, and each of these was well marked in my patient.

To this day half of her friends, from her uncertain gait upon the street and her peculiarities of moods, her difficulties with servants, believe the latter years of her life to have been clouded with the habit of daily intoxication, while I know perfectly well that during these years she was an almost total abstainer.

After having been under my care for rather more than a year, and after being confined to her bed and her chair with a sprained ankle for several weeks, she was found one morning with marked right hemiplegia and unconsciousness.

During the forenoon the mind cleared to the extent of her making signs with the left hand and attempts at speech.

This improvement was continuous, and in the course of a week the mind was perfectly clear, the hemiplegia was complete, but she could only say "yes" and "no," and that not always at the right time, apparently only said the words automatically, and did not know which word she had used. In a short time the word "no" was lost, and "yes" was her entire vocabulary.

During the early weeks of her sickness she was much tormented with something she wished to communicate, and her fruitless attempts were simply agonizing, and I now recall them as the severest ordeals of my professional experience.

During this time, and in fact during her whole remaining life, the language of gesture was never for a moment at fault. The left hand, the head, the muscles of the face, and the tone of the voice never for a moment failed to express her thoughts, and as she grew better her nurses, attendants and myself often remarked that words were not of much importance, that there was a great deal of language without talking.

From the first recovery of consciousness, a few hours after the attack, we saw that she understood everything said in her presence, and this ability remained up to the time of the subsequent and fatal seizure, nearly a year from the date of her first attack. During this time certain observations were made upon the state of her faculties, of considerable scientific interest.

As I said, for weeks during the earlier part of the illness, she was almost wild with the desire to make some communication to us, and particularly to me, and in my attempts to find what she wanted to say, to assist her in making her wants known, I demonstrated that she had not the slightest memory of the form, construction or use of words. She could not find on a card, with the letters of the alphabet printed plainly, the letters to spell the words "yes" or "no," or, in fact, any word of the language. More than this, I could not teach her the simplest word, although she tried again and again, with all the earnestness of desperation, to see and follow the steps by which the word was composed. I remember at the time that it was like telling one, in absolute darkness, to see. The mind could not take the first step in the desired direction. During this whole time there was not the first sign of mental impairment in any other direction. Papers, magazines and books were daily read to her, and we talked over all the topics of the day, whether they were local, national or even of a wider interest. Her memory was never for a moment at fault, and she continued to be the imperious mistress of her house, as she had ever been.

The aphasia came early in the month of October of the year of our presidential election, and we soon learned that the illustrated papers were as plain and intelligible to her as to any one; she saw them regularly, and at once recognized the prominent political characters as they, from time to time, appeared, and was plainly perfectly appreciative of the situations in which they were intended to be placed.

The case terminated in the month of August of the next season, and was accompanied with complete paralysis of the left side, the right being previously paralyzed, which was succeeded by loss of consciousness on the third day and death on the fifth.

Aside from the interest this case had from the clinical point of view, there was to me a question of considerable interest in the situation of one in full mental health, cut off from communicating with one's friends, by the means of spoken or written words, and the thought would come to me, again and again, that our science should have a remedy for the painful situation. The language of signs should be so developed as to be available in such cases. Before my patient's death we had constructed quite an extensive vocabulary of signs, and thereby made communication quite easy and reliable.

Aphasia is usually treated as a disorder of speech, but would be more properly regarded as a disorder of memory, and the chief interest to the profession arises from the light which its study has thrown upon that faculty of the mind, and I would, for a few moments, direct your attention to the present views of that faculty. In the use of the term "memory a faculty of the mind," I am simply following the custom of early days, and I bring the term to your attention for the purpose of pointing out a certain chance in the term for a misconception. Following the thought of memory as a faculty of the mind; students were wont to give that faculty a personality, an entity, and also to locate the same in some definite part of the brain as its seat; to consider the faculty as something complete, concrete. This tendency is illustrated by the custom of saying that a person had a good or a poor memory, or that such â one had lost or regained his memory. This was the habit of our early teachers; whereas, we are now taught that memory is a term applied to the action of nerve tissues; that the phenomena of memory is one of the most distinctive exhibitions of nerve action; that memory is a mode of nerve action, and that it consists in the property of nerve tissues of receiving impressions, of storing those impressions, as it were of registering impressions and of reproducing those impressions. I think that you will at once see that this is quite an expansion of our thought.

Instead of memory being a faculty of the mind by which we remember the doses of certain drugs, the signs of certain diseases, the directions and restrictions of our code of ethics, it becomes a property of our organization by which we recognize the difference between ourselves and that which is not ourselves; that instead of memory being concrete and simple, it is complex and multiple; that instead of one having a memory, he has as many memories as he has senses; that we have a visual memory, an acoustic memory, a gustatory memory, an olfactory memory, a tactile memory; and that each of these is but a family comprising many individuals.

I can not detain you with even the bare enumeration of the distinct memories, whose accumulations we are using at each hour of our lives, but I will say, that it is the teaching of the literature of the day that each of these memories has a definite seat in the brain; that the acquirements or residua of each has a mechanical, a chemical, a nutritional exponent or counterpart in the brain; that each memory has its cerebral organ; that the normal performance of the function of each depends upon normal cerebral nutrition, and that from abnormal circulation and consequent deranged nutrition, the function of each memory may be perverted or arrested.

Clinical medicine gives abundant testimony to show the localization, the individuality and the independence of the cerebral organs of the various memories, and furnishes numerous instances of the loss of one or more of these organs, while the others remain in regular and normal action.

In fact, this advance in cerebral physiology has been made by using the materials furnished by the clinical physician and the pathologist, and of these materials the phenomena of aphasia have been among the most interesting and the most important.

This reminds me that this is supposed to be a paper upon aphasia, and that being the case, you will naturally expect me to say something upon that subject, and as I have carefully avoided the topic up to this time, we will at least remark in passing that aphasia is not a disease, but a symptom, and that essentially it shows itself as an impairment or a loss of the power to express one's thoughts by use of the faculty of speech.

Clinically the symptoms vary in the most marked degree. Aphasia may exist as a slight loss of readiness of speech, a liability to forget a frequently used word, as one's own name, or the name of an intimate friend, or may be so extensive as to forbid the use of any words, or even signs or gestures, at the same time thought is normally present. Then again, one can speak but cannot write, or can write but cannot speak. Cases are also reported where the reception of words was similarly interfered with. One could not understand spoken words, but could written ones, and *vice versa.*

Ataxic aphasia, amnesic paraphasia, agrammatism, agraphia, word-deafness, word-blindness, are terms used to denote the particular memory whose impairment or loss constitutes the degrees or varieties of aphasia, or, as we should say, disease.

You will also expect me to refer to the theory that the seat of the lesion resulting in aphasia is in the region of the third convolution of the frontal lobe, or in the island of Reil or its neighborhood, and also to the coincidence of aphasia and right-sided hemiplegia, as well as the fact that left-handed people get the disease with left-sided paralysis.

These facts have been frequently observed, and freely commented upon, and will doubtless receive, in your discussion, such attention as their interest and importance demand. My purpose will be served if in this connection you keep before your minds the facts concerning the nature of memory which the study of aphasia has helped to establish:

1. That memory is a faculty common to all sentient beings.

2. That it is by the possession of this faculty that such beings conserve and reproduce the impressions caused by the forces of the external world.

3. That the operation of each one of our senses is through the medium of several distinct and independent memories.

4. That each of these different memories has its seat in a definite portion of the brain.

5. In fact, that every remembered thing, fact or impression, has its seat and counterpart in the brain, just as the photographer preserves the negatives of the pictures he has taken.

6. That from disease these registrations may be permanently or temporarily destroyed, and the individual thus lose single facts or whole groups or classes of such facts.

7. That the memory of a single idea, that is, of the thought which is in the mind before the spoken word is, and to express which the spoken word is used, and the memory of the motor combination by which we speak the word, and also the memory of the motor combination by which we write the word, as well as the memory of the acoustic combination by which we understand this word when spoken to us, and the visual memory by which we correctly interpret the same when we see it written or printed; that each of these memories of a given idea occupy separate locations in the brain, and that some may be lost without the impairment of the others.

Let us return to the case of Mrs. F. and see what was the condition as to the loss or the preservation of her memories.

In so far as we could ascertain, the world at large was the same to her after the attack as before. Horses were horses, dogs were dogs, friends were friends; everything in her house was recognized at sight, and she had forgotten nothing that she had ever seen, or heard, touched, smelled or tasted. She knew everything, where everything was, and what everything was for, but knew the name of nothing. The memories by which the various senses brought her in contact with her environment were preserved intact, but the motor combinations or memories by which these various impressions were represented in characters of vocal speeeh, or the characters of written speech, were utterly destroyed.

For instance, in a given case, for example, the substance water. She retained her memory of its appearance and sight, and also its touch and its taste. She knew its uses to bathe in and to drink of when thirsty. She also recognized its name when we used the word, as in asking if she would have a glass of water. But how to go to work to produce a sound which should be that which we hear in the word "water," she had no more idea of than one has of vision without light. Language made the same impression upon her that it ever had, but she had no memory of the use of words as the signs of ideas.

This was not from lack of ability to vocalize, for she was habitually engaged in this exercise, and would express a wide range of feeling and thought by vocal sounds, but always without even an attempt at the formation of words. This state seemed to be one of permanence; there was no improvement from the beginning of the attack until the end. In the use of gesture there was never any uncertainty or confusion, and as time passed the nurse had no difficulty in reading her signs, and she ordered her meals, her horses; she inquired after her friends, and sent messages to them with as much directness as if in full possession of the faculty of speech.

The interest in this case and subject seems to be from the light thrown upon the processes of the mind, and the result is to indicate that in regard to memory, the apparently simple is quite complex and multiform; that memory is primarily a biological fact, and may exist with or without consciousness, and that its consideration is entirely within the domain of cerebral physiology.

